# A 29 Mainland Chinese cohort of patients with Phelan–McDermid syndrome: genotype–phenotype correlations and the role of *SHANK3* haploinsufficiency in the important phenotypes

**DOI:** 10.1186/s13023-020-01592-5

**Published:** 2020-11-30

**Authors:** Na Xu, Hui Lv, Tingting Yang, Xiujuan Du, Yu Sun, Bing Xiao, Yanjie Fan, Xiaomei Luo, Yongkun Zhan, Lili Wang, Fei Li, Yongguo Yu

**Affiliations:** 1 Department of Pediatric Endocrinology and Genetic Metabolism, Shanghai Institute for Pediatric Research, Shanghai Key Laboratory of Pediatric Gastroenterology and Nutrition, Xinhua Hospital, School of Medicine, Shanghai Jiao Tong University, Room 801, Science and Education Building, Kongjiang Road 1665, Shanghai, 200092 China; 2 Department of Developmental and Behavioral Pediatrics, Department of Child Primary Care, Brain and Behavioral Research Unit of Shanghai Institute for Pediatric Research and MOE-Shanghai Key Laboratory for Children’s Environmental Health, Xinhua Hospital, School of Medicine, Shanghai Jiao Tong University School of Medicine, Kongjiang Road 1665, Shanghai, 200092 China; 3 Shanghai Key Laboratory of Pediatric Gastroenterology and Nutrition, Shanghai, 200092 China

**Keywords:** Phelan–McDermid syndrome (PMS), Mainland China, *SHANK3* haploinsufficiency, Genotype–phenotype correlation

## Abstract

**Background:**

Phelan–McDermid syndrome (PMS) or 22q13 deletion syndrome is a rare developmental disorder characterized by hypotonia, developmental delay (DD), intellectual disability (ID), autism spectrum disorder (ASD) and dysmorphic features. Most cases are caused by 22q13 deletions encompassing many genes including *SHANK3*. Phenotype comparisons between patients with *SHANK3* mutations (or deletions only disrupt *SHANK3*) and 22q13 deletions encompassing more than *SHANK3* gene are lacking.

**Methods:**

A total of 29 Mainland China patients were clinically and genetically evaluated. Data were obtained from medical record review and a standardized medical history questionnaire, and dysmorphology evaluation was conducted via photographic evaluation. We analyzed 22q13 deletions and *SHANK3* small mutations and performed genotype–phenotype analysis to determine whether neurological features and other important clinical features are responsible for haploinsufficiency of *SHANK3*.

**Results:**

Nineteen patients with 22q13.3 deletions ranging in size from 34 kb to 8.7 Mb, one patient with terminal deletions and duplications, and nine patients with *SHANK3* mutations were included. All mutations would cause loss-of function effect and six novel heterozygous variants, c.3838_3839insGG, c.3088delC, c.3526G > T, c.3372dupC, c.3120delC and c.3942delC, were firstly reported. Besides, we demonstrated speech delay (100%), DD/ID (88%), ASD (80%), hypotonia (83%) and hyperactivity (83%) were prominent clinical features. Finally, 100% of cases with monogenic *SHANK3* deletion had hypotonia and there was no significant difference between loss of *SHANK3* alone and deletions encompassing more than *SHANK3* gene in the prevalence of hypotonia, DD/ID, ASD, increased pain tolerance, gait abnormalities, impulsiveness, repetitive behaviors, regression and nonstop crying which were high in loss of *SHANK3* alone group.

**Conclusions:**

This is the first work describing a cohort of Mainland China patients broaden the clinical and molecular spectrum of PMS. Our findings support the effect of 22q13 deletions and *SHANK3* point mutations on language impairment and several clinical manifestations, such as DD/ID. We also demonstrated *SHANK3* haploinsufficiency was a major contributor to the neurological phenotypes of PMS and also responsible for other important phenotypes such as hypotonia, increased pain tolerance, impulsiveness, repetitive behaviors, regression and nonstop crying.

## Introduction

Phelan–McDermid syndrome (PMS, OMIM 606232), also called 22q13 deletion syndrome is a rare developmental disorder with diverse clinical features [1–4]. Given the heterogeneity of genetic etiology, patients diagnosed as PMS usually comorbid with multi disorders, such as neural system related disorders (severely delayed speech, DD/ID, ASD, and seizures, et al.), recurring upper respiratory tract infections, gastroesophageal reflux, and minor dysmorphic features (dysplastic ears, bulbous nose, and long eyelashes, et al.) [5, 6].

PMS is caused by deletions ranging from hundreds of kilobases (kb) to over nine megabases (Mb) in size in the 22q13 region [7]. Chromosomal abnormalities include simple terminal deletions, interstitial deletions, translocations and ring chromosomes. Almost all individuals identified to date involve *SHANK3*, mapping to the distal end of 22q13.33 [1, 8–11]. *SHANK3* encodes a scaffolding protein enriched in the postsynaptic density of glutamatergic synapses and play a critical role in synaptic function and dendrite formation [12, 13]. Deletions or point mutations in *SHANK3* have been identified in patients ascertained for ASD at a rate of about 2% [14], intellectual disability (ID) at a rate of about 2% [15], and schizophrenia at a rate of 0.6–2.16% [16, 17].

Currently, the hypothesis is that *SHANK3* haploinsufficiency is responsible for major neurological features of PMS [8, 18, 19]. Wilson and colleagues detected few correlations between the deletion size and the most neurological features. They have also reported correlations between deletion size and other important phenotypes including hypotonia, head circumference, recurrent ear infections, pointed chin, and dental anomalies [19]. In addition, genotype–phenotype correlation analyses suggest that the size of deletion is a predictor of phenotypic severity. Specifically, developmental delay [19–21], hypotonia [19–21], dysmorphic features [5, 21, 22], language status [22, 23], social communication deficits related to ASD [5, 22], renal abnormalities [5, 22], lymphedema [5, 22], seizures [5] show a higher incidence or increased severity with larger deletions. However, genotype–phenotype correlation analyses have largely focused on patients with 22q13 deletions, only two reports on PMS have contained a few patients carrying *SHANK3* mutations or deletions only disrupt *SHANK3* [5, 22]. Silvia De Rubeis and colleagues compared clinical features in individuals with *SHANK3* mutations to 22q13 deletions including *SHANK3* from the literature. However, they did not analyze genotype–phenotype correlations. The lack of genotype–phenotype correlations between patients with *SHANK3* mutations (or deletions only disrupt *SHANK3*) and patients with deletions encompassing more than *SHANK3* gene have hindered the exploration of *SHANK3* deficiency in the important phenotypes in PMS.

Over the past few years, more than 1400 cases were identified worldwide. However, thus far, only four 22q13 deletions and a *SHANK3* mutation in patients with ID have been reported in Mainland China [24]. Here, we report 29 previously undescribed Mainland Chinese patients with PMS. Our main goal was to explore the contribution of *SHANK3* haploinsufficiency to the important phenotypes besides neurological features. We also presented clinical profile and genetic spectrum of the patients, aiming at expanding the molecular and phenotypic spectrum of PMS.

## Results

### Characterization of 22q13.3 deletions and *SHANK3* mutations in PMS patients

**Fig. 1:**
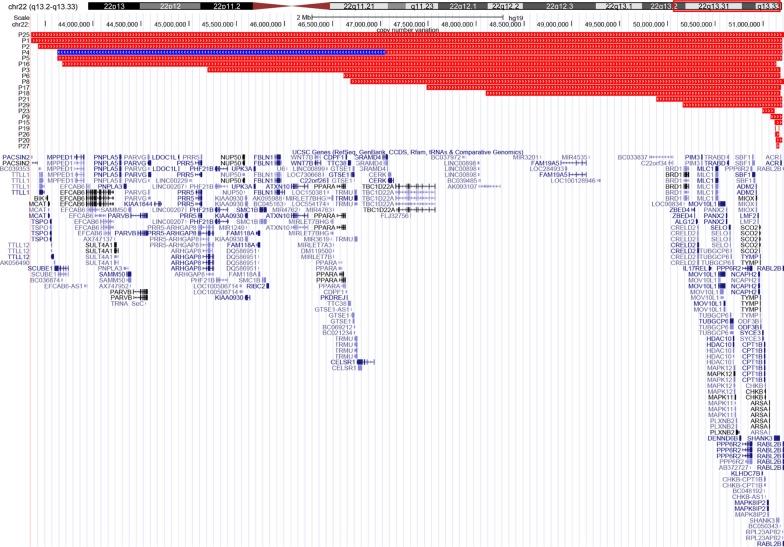
Distribution of deletions and duplications among 20 China mainland patients that varied from 34 kb to 8.7 Mb

**Fig. 2:**
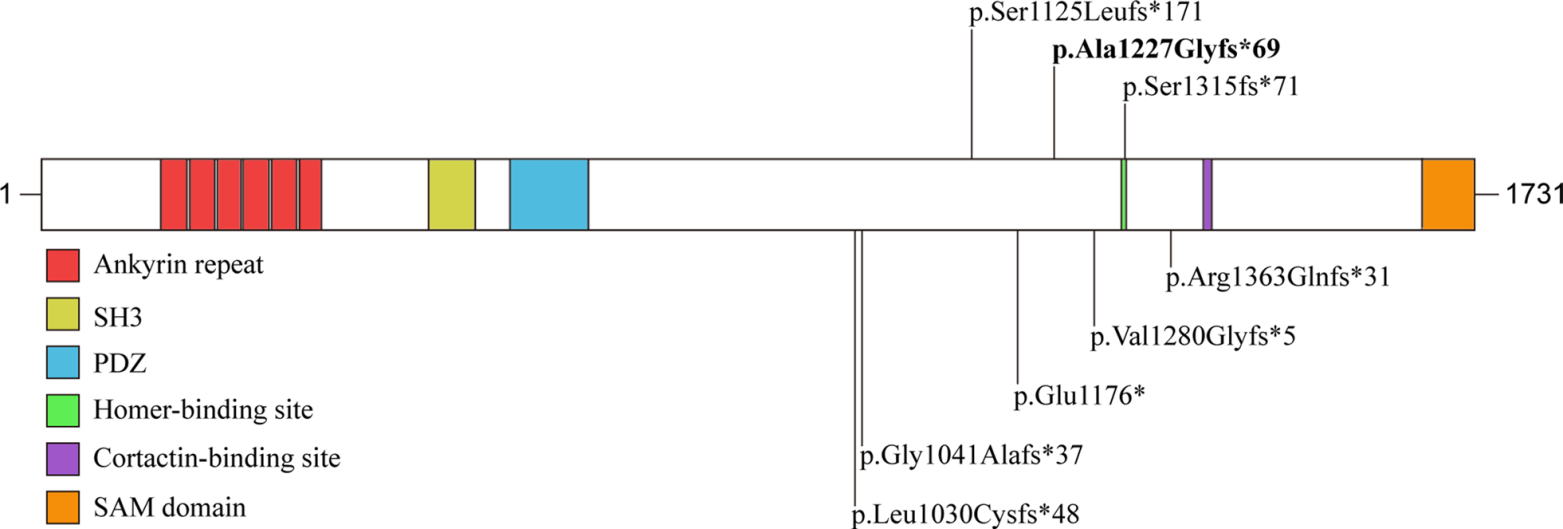
Distribution of *SHANK3* mutations in our cases. Recurrent mutations are indicated in bold. Protein domains are from UniProt; the homer and cortactin binding sites are indicated as previously reported [2]

We reported 29 children identified by a custom 22q13 microarray or Whole exome sequencing (WES). Nineteen children carried 22q13 terminal deletions and one child harbored terminal deletions accompanied by proximal duplications (Table 1 and Fig. 1). The deletions ranged from 34 kb to 8.7 Mb with a mean size of 3617 kb. Three children had small deletions ranging from 34 to 57 kb in size that only contain *SHANK3* gene. Nine children with *SHANK3* mutations tested through WES were also observed (Table 2 and Fig. 2). The variants were named following Human Genome Variation Society nomenclature guideline. As reported previously [25], the human genome reference assembly (GRCh37/hg19) misses the beginning of exon 11, nucleotide and amino acid positions were corrected according to the *SHANK3* mRNA (NM_033517.1) and protein (NP_277052.1) in RefSeq. The variants included eight frameshift and one nonsense mutations (Table 2, Fig. 2). Remarkably, we found a recurrent frameshift mutation, c.3679dupG (p. Ala1227Glyfs*69) in two unrelated patients. The mutations arose de novo in nine patients. Six variants caused loss of the Homer-binding, the Cortactin-binding and the Sterile Alpha Motif (SAM) domains. One variant disrupted Homer-binding domains and caused loss of the Cortactin-binding and SAM domains. And one variant caused loss of the Cortactin-binding and the SAM domains (Fig. 2). Besides, we assessed pathogenicity of *SHANK3* mutations in our cohort. Variants listed in Table 2 met the following criteria: (1) loss-of-function variants (frameshift and nonsense, splice site), (2) De novo (both maternity and paternity confirmed) in a patient with the disease and no family history, and (3) absent from control databases (EVS and gnomAD). Except for one variant (c.3942delC, p.Gly1041Alafs*37) was likely pathogenic, the rest variants were pathogenic according to the ACMG variant interpretation guidelines [26]. Six novel heterozygous variants, c.3838_3839insGG, c.3088delC, c.3526G > T, c.3372dupC, c.3120delC and c.3942delC, were reported.

**Table1:** Details of the 22q13.3 deletions in 20 individuals with PMS

Patient	Ascertainment method	Rearrangement	Array coordinates (hg19)	Deletion or duplication size (kb)	Genes	Inheritance	Other chromosome
P1	SNP	Deletion	2,243,352,384–51,197,766	7987	Many	NK	None
P2	SNP	Deletion	43,428,841–51,197,766	7956	Many	De novo	None
P3	aCGH	Deletion	45,193,180–51,178,213	6042	Many	De novo	None
P4	SNP	Duplication and	(43,199,808–47,056,500) × 3;	3891	Many	NK	None
		Deletion	47,056,555–51,197,766	4044	Many		
P5	SNP	Deletion	43,629,482–51,197,766	7568	Many	NK	None
P6	aCGH	Deletion	46,615,777–51,178,213	4710	Many	NK	None
P8	SNP	Deletion	46,688,687–51,220,738	4426	Many	NK	None
P9	SNP	Deletion	51,073,379–51,197,838	124	Many	NK	None
P15	SNP	Deletion	51,121,363–51,197,766	76	*SHANK3, ACR*	De novo	None
P16	SNP	Deletion	43,674,432–51,139,778	7649	Many	NK	None
P17	SNP	Deletion	47,486,331–51,183,840	3698	Many	NK	None
P18	SNP	Deletion	48,100,752–51,177,162	3004	Many	NK	None
P19	QPCR	Deletion	51,121,768–51,121,840; 51,158,612–51,160,865	57	*SHANK3*	De novo	None
P20	SNP	Deletion	51,128,324–51,197,766	69	*SHANK3, ACR*	NK	None
P21	SNP	Deletion	49,882,209–51,197,726	1316	Many	NK	None
P23	SNP	Deletion	50,990,475–51,115,526	125	Many	NK	None
P25	WES	Deletion	42,522,566–51,216,419	8909	Many	NK	None
P26	MLPA	Deletion	51,123,013–51,169,790	46	*SHANK3*	NK	None
P27	SNP	Deletion	51,135,991–51,169,740	34	*SHANK3*	NK	None
P29	SNP	Deletion	50,155,448–51,197,766	1042	Many	NK	None

*NK* not known, *WES* whole exome sequencing, *aCGH* array-CGH, *SNP* single nucleotide polymorphism, *MLPA* multiplex ligation-dependent probe amplification, *QPCR* quantitative real-time PCR

**Table 2:** Summarization of *SHANK3* gene variants in 9 patients with PMS

Patient	Variant location^a^	Variant type	Protein change	Inheritance	Literature report	MutationTaster	ACMG classification
P7	c.3120delC	Frameshift	p. Gly1041Alafs*37(Het)	NK	–	Disease causing	Likely pathogenic
P10	c.3372dupC	Frameshift	p. Ser1125Leufs*171(Het)	De novo	–	Disease causing	Pathogenic
P11	c.3526G > T	Nonsense	p. Glu1176*(Het)	De novo	–	Disease causing	Pathogenic
P12	c.4086_4087delAC	Frameshift	P. Arg1363Glnfs*31(Het)	NK	Nature. 2017;542(7642):433–438	Disease causing	Pathogenic
P13	c.3679dupG	Frameshift	p. Ala1227Glyfs*69(Het)	De novo	Nat Genet. 2007;39(1):25‐27	Disease causing	Pathogenic
P14	c.3679dupG	Frameshift	p. Ala1227Glyfs*69(Het)	NK	Nat Genet. 2007;39(1):25‐27	Disease causing	Pathogenic
P22	c.3088delC	Frameshift	p. Leu1030Cysfs*48(Het)	De novo	–	Disease causing	Pathogenic
P24	c.3942delC	Frameshift	p. Ser1315fs*71(Het)	De novo	–	Disease causing	Pathogenic
P28	c.3838_3839insGG	Frameshift	p. V1280Gfs*5(Het)	De novo	–	Disease causing	Pathogenic

*NK* not known, * the stop codon, *ACMG* American College of Medical Genetics and Genomic

^a^NM_033517.1

^b^NP_277052.1

### Overall prevalence of clinical phenotype

The most common phenotype seen in our cases (Tables 3, 4 and Additional file 1: Table S1) were speech delay (100%), DD/ID (88%), ASD (80%), hypotonia (83%), distinctive behavioral abnormalities such as hyperactivity (83%), impulsiveness (76%) and repetitive behaviors (69%), developmental/neurological abnormalities such as increased pain tolerance (62%) and gait abnormalities (55%). Other common features included any type of seizures (24%), chewing difficulties (31%), biting self or others (30%), hair pulling (31%), aggressive behavior (30%), nonstop crying (45%), sleep disturbance (24%), diarrhea/constipation (23%) and eczema (28%), and minor dysmorphic traits included descending palpebral fissure (33%), ear anomalies (24%), periorbital fullness (24%), strabismus (29%) and frontal bossing (19%).

**Table 3:** Developmental and behavioral features of patients with PMS in our cohort as compared to the literature

Feature	N	Total	%	Literature frequency^a^ (%)
Developmental/neurological				
Speech (age ≥ 3)				
Absent speech	11	20	55	
Sentences	9	20	45	
Walk independently (age ≥ 3)	20	20	100	
DD/ID	23	26	88	
Any type of seizures (including febrile seizures)	7	29	24	14–41
Overheats or turns red easily	5	29	17	68^b^
Decreased perspiration	2	29	7	60^b^
Overly sensitive to touch	5	29	17	46^b^
Increased pain tolerance	18	29	62	29–100
Arachnoid cyst	4	29	14	19^b^
Hypotonia	24	29	83	29–100
Gait abnormalities	16	29	55	93^c^
Behavioral features				
Autism spectrum disorder (age ≥ 3)	16	20	80	31^b^
Impulsiveness	22	29	76	47^c^
Chewing difficulties (difficulty with eating)	9	29	31	58^c^
Biting (self or others)	9	29	30	46^b^
Hair pulling	9	29	31	41^b^
Excessive screaming	5	29	17	31^b^
Aggressive behavior	9	29	30	28^b^
Nonstop crying (crying non-stop for 3 h)	13	29	45	21^b^
Hyperactivity	24	29	83	47^c^
Self-injury	3	29	10	
Pica	3	29	10	
Repetitive behaviors	20	29	69	100^c^
Sleep disturbance	7	29	24	41–46
Regression	13	29	45	65^d^
Other clinical features				
Gastroesophageal reflux	2	29	7	> 25–44
Diarrhea/constipation	7	29	23	38–41
Congenital heart defect	4	29	14	3^c^
Genital anomalies	1	23	4	5^b^
Eczema	8	29	28	18^d^
Enzyme deficiency	0	29	0	4^b^
Immune deficiency	1	29	3	12^b^
Recurring upper respiratory tract infections	6	29	21	29–100
Hearing loss	1	29	3	3^d^
Allergies	5	29	17	
Asthma	1	29	3	
Renal abnormalities	3	21	14	17–38
Lymphedema	0	29	0	22–29
Hypothyroid	0	29	0	6^b^
Diabetes	0	29	0	2^b^
Vitiligo	0	29	0	3^c^

^a^Frequencies based on the literature review available [27]

^b^Frequencies available in the clinical and genomic evaluation of 201 patients with PMS [23]

^c^Frequencies based on a Brazilian cohort of individuals with PMS [22]

^d^Frequency based on the analysis of PMS individuals carrying *SHANK3* point mutations [28]

^e^Frequencies based on a previous report [5]

**Table 4:** Dysmorphic features in patients with Phelan–McDermid syndrome

Dysmorphic features	N	Total	%	Literature frequency^a^ (%)
Microcephaly^c^	2	20	10	13
Macrocephaly^d^	1	20	5	7
Sparse eyebrows	3	21	14	18^b^
Long eyelashes	1	21	5	45
Periorbital fullness	5	21	24	45
Descending palpebral fissure	7	21	33	
Hypertelorism	3	21	14	36
Strabismus	6	21	29	26
Epicanthal folds	1	21	5	57
Wide nasal bridge	3	21	14	16
Large/wide nose	2	21	10	
Bulbous nose	3	21	14	80
Anteverted nares	1	21	5	9^b^
Full cheeks	1	21	5	25
High forehead	4	21	19	
Short philtrum	1	21	5	0^b^
Ear anomalies	5	21	24	54
Thick lower vermillion	3	21	14	27^b^
Down-turned mouth	3	21	14	
Short stature/delayed growth^e^	1	29	3	11
Tall stature/accelerated growth^f^	5	29	17	9

^a^Frequencies based on the literature review available [27]

^b^Frequency based on the analysis of PMS individuals carrying *SHANK3* point mutations [28]

^c^Head circumference < 3rd percentile

^d^Head circumference > 98th percentile

^e^Height < 3rd percentile

^f^Height > 98th percentile

### Development, language skills and neurological features

DD/ID was evaluated by analyzing the patients’ medical records or questionnaire (n = 26). Four patients were severe to extremely severe and nine patients were mild to moderate. Eleven cases were diagnosed as DD/ID but we were not able to assess the degree of intellectual disability, and two showed normal intelligence (Tables 3, 4 and Additional file 1: Table S1).

All individuals (21/20, 100%) could walk independently (Table 3 and Additional file 1: Table S1) by 3 years of age, although fine and gross motor delays could be observed in all individuals at the age of test. The mean age when individuals acquired this skill was 20 months, ranging from 12 to 33 months of age. Only three patients (P1, P5 and P9) had ever been received rehabilitation therapy by 3 years of age. The majority of individuals showed hypotonia (24/29, 83%) and gait abnormalities (16/29, 55%).

Language delay was notable (20/20, 100%) over the age of 3 years (Results are summarized in Table 3). In total, 52% of the cases (11/21) had no speech, 9 had “sentences” (spoke single word or spoke in phrases or sentences). The degree of language delay in ten patients ranged from mild to moderate and seven patients ranged from severe to extremely severe.

Seven participants (7/29, 43%) demonstrated seizures (Table 2). Abnormal electroencephalography (EEG) were described in six participants (6/18, 33%), including three without clinical seizures. MRI showed abnormal findings in fourteen participants (13/21, 62%), including delayed myelination (n = 2), abnormal corpus callosum (n = 5), enlargement of ventricles (n = 2), white matter abnormalities (n = 1), generous extracerebral spaces (n = 1) and large cisterna magna (n = 2).

### Behavioral features

By the age of 3 years, 80% patients (16/20) was diagnosed as ASD. As listed in Table 3, the majority of patients were reported as hyperactivity (24/29, 83%), impulsiveness (22/29, 76%) and repetitive behaviors (20/29, 69%) such as hand flapping, teeth grinding. Patients were also prone to nonstop crying (13/29, 45%) and regression (13/29, 45%) affecting language, cognitive and motor ability.

### Other clinical features

According to parents’ report, the majority of participants had increased pain tolerance (18/29, 62%). In addition, chewing difficulties (9/29, 31%), eczema (8/29, 28%) diarrhea/constipation (7/29, 23%), renal problems (3/21, 14%) and recurring upper respiratory tract infections (6/29, 21%) are common. Similarly, hearing loss reported in 3% of cases with PMS, were uncommon [27]. Lymphedema, hypothyroid, diabetes, vitiligo and enzyme deficiency have been reported in patients with PMS [5, 22, 23, 27], but were absent in individuals in our cases (Table 3).

### Dysmorphic features

Dysmorphology examinations were performed in 21 patients by two clinicians. They all had at least one feature (Table 4 and Additional file 1: Table S2). Dysmorphic features in our cohort were not as so common as reported in previous studies [5, 22, 23]. The most frequent features in this study were ear anomalies (5/21, 24%), descending palpebral fissure (7/21, 33%), periorbital fullness (5/21, 24%), strabismus (6/21, 29%), frontal bossing (4/21, 19%).

### *SHANK3 *mutations (or deletions only disrupt *SHANK3*) effects

**Fig. 3:**
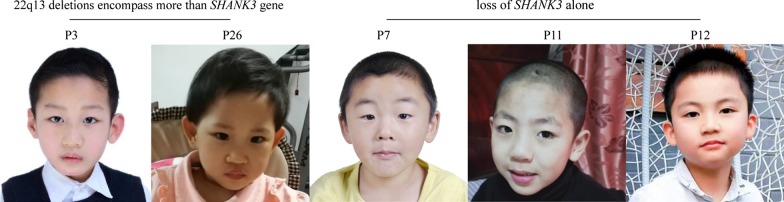
Representative images of patients with PMS showing mild dysmorphism. There was no significant difference between patients with 22q13 deletions encompassing more than *SHANK3* gene and patients with loss of *SHANK3* alone in dysmorphic features

To investigate the role of *SHANK3* haploinsufficiency in the important phenotypes, we divided patients into two groups. Group 1 (n = 17) included individuals with deletions that encompass more than *SHANK3* gene. Group 2 (n = 12) consists of individuals with *SHANK3* mutations or deletions that only encompass *SHANK3*. As shown in Table 5, the frequency of the hypotonia in patients in the group 2 was higher compared to that in the group 1 (100% vs. 71%), with statistical significance near the borderline (Fisher’s exact two-sided test *P* = 0.059), suggesting the haploinsufficiency for *SHANK3* may contribute to the hypotonia. There was no significant difference between group 1 and group 2 in the frequency of other clinical features including DD/ID (10/11, 91%), ASD (7/8, 88%), increased pain tolerance (7/12, 58%), gait abnormalities (6/12, 50%), impulsiveness (11/12, 92%), repetitive behaviors (9/12, 75%), regression (7/11, 63%), nonstop crying (7/12, 58%) and minor dysmorphic traits including ear anomalies (4/10, 40%) and descending palpebral fissure (3/10, 30%) which are high in loss of *SHANK3* alone group (Table 5). In addition, as shown in Fig. 3, children in group 1 did not show more severe dysmorphic traits compared to children in group 2. The minor dysmorphic traits the patients in Fig. 3 had were listed as followings: Patient 3 had short philtrum and down-turned mouth, patient 25 had long eyelashes and down-turned mouth, patient 7 had a descending palpebral fissure, strabismus and wide nasal bridge, patient 11 had a bulbous nose and fleshy ears and patient 12 had low set ears.

**Table 5:** Comparison of the prevalence of phenotypes between patients with 22q13 deletions encompassing more than *SHANK3* gene and loss of *SHANK3* alone

Clinical feature	22q13 deletions encompass more than *SHANK3* gene	Loss of *SHANK3* alone	*P* value^a^
Physical features			
Microcephaly^b^	2/11(18%)	1/10(10%)	1.000
Macrocephaly^c^	0/11(0%)	1/10(10%)	1.000
Sparse eyebrows	1/11(9%)	2/10(20%)	0.586
Long eyelashes	0/11(0%)	1/10(10%)	1.000
Periorbital fullness	3/11(27%)	2/10(20%)	1.000
Descending palpebral fissure	4/11(36%)	3/10(30%)	1.000
Hypertelorism	3/11(27%)	0/10(0%)	0.214
Strabismus	4/11(36%)	2/10(20%)	0.635
Epicanthal folds	0/11(0%)	1/10(10%)	1.000
Wide nasal bridge	2/11(18%)	1/10(10%)	1.000
Large/wide nose	2/11(18%)	0/10(0%)	1.000
Bulbous nose	1/11(9%)	2/10(20%)	1.000
Anteverted nares	0/11(0%)	1/10(10%)	1.000
Full cheeks	0/11(0%)	1/10(10%)	1.000
Frontal bossing	2/11(18%)	2/10(20%)	1.000
Short philtrum	1/11(9%)	0/10(0%)	1.000
Ear anomalies	1/11(9%)	4/10(40%)	0.149
Thick lower lip	1/11(9%)	2/10(20%)	0.586
Down-turned mouth	2/11(18%)	1/10(10%)	1.000
Short stature/delayed growth^d^	1/17(6%)	1/12(8%)	1.000
Tall stature/accelerated growth^e^	4/17(24%)	1/12(8%)	0.370
Developmental/neurological			
DD/ID	13/15(87%)	10/11(91%)	1.000
Speech (absent speech versus sentences, age ≥ 3)	8/12(67%)	3/8(38%)	0.370
Any type of seizures (including febrile seizures)	4/17(24%)	3/12(25%)	1.000
Overheats or turns red easily	3/17(13%)	2/12(17%)	1.000
Decreased perspiration	2/17(6%)	0/12(0%)	1.000
Overly sensitive to touch	3/17(18%)	2/12(17%)	1.000
Increased pain tolerance	11/17(65%)	7/12(58%)	1.000
Arachnoid cyst	3/17(18%)	1/12(8%)	0.622
Hypotonia	12/17(71%)	12/12(100%)	0.059
Gait abnormalities	10/17(59%)	6/12(50%)	1.000
Neuroimaging abnormalities	9/12(75%)	4/9(44%)	0.159
Behavioral features			
Autism spectrum disorder (age ≥ 3)	10/17(59%)	7/8(88%)	0.205
Impulsiveness	11/17(65%)	11/12(92%)	0.187
Chewing difficulties (difficulty with eating)	5/17(29%)	5/12(42%)	0.694
Biting (self or others)	5/17(29%)	4/12(33%)	1.000
Hair pulling	4/17(19%)	5/12(42%)	0.231
Excessive screaming	3/17(24%)	2/12(17%)	1.000
Aggressive behavior	5/17(29%)	4/12(33%)	1.000
Nonstop crying (crying non-stop for 3 h)	6/17(35%)	7/12(58%)	0.274
Hyperactivity	14/17(82%)	10/12(83%)	1.000
Self-injury	3/17(24%)	0/12(0%)	0.246
Pica	1/17(6%)	2/12(17%)	0.553
Repetitive behaviors	11/17(65%)	9/12(75%)	0.694
Sleep disturbance	4/17(19%)	3/12(25%)	1.000
Regression	5/17(29%)	7/11(63%)	0.121
Other clinical features			
Gastroesophageal reflux	2/17(12%)	0/12(0%)	0.498
Diarrhea/constipation	3/17(24%)	4/12(33%)	1.000
Genital anomalies	1/15(7%)	0/7(0%)	1.000
Eczema	4/17(24%)	4/12(33%)	0.683
Immune deficiency	1/17(7%)	0/12(0%)	1.000
Recurring upper respiratory tract infections	5/17(29%)	1/12(8%)	0.354
Hearing loss	1/17(7%)	0/12(0%)	1.000
Congenital heart defect	2/17(12%)	1/12(8%)	1.000
Allergies	3/17(24%)	2/12(17%)	1.000
Asthma	1/17(7%)	0/12(0%)	1.000

^a^All *P* values are Fisher’s Exact

^b^Head circumference < 3rd percentile

^c^Head circumference > 98th percentile

^d^Height < 3rd percentile

^e^Height > 98th percentile

## Discussion

Up to now, only four cases with 22q13 deletions or a *SHANK3* mutation were reported in a cohort of patients with ID in Mainland China [24]. To fill in the gap, we presented the 29 participants with PMS in Mainland China to broaden the clinical and genetic spectrum of PMS especially in Chinese ethic group.

Findings in our cases indicate *SHANK3* mutations are fully penetrant in accordance with previous estimates [28]. Notably, seven out of eight variants lead to the loss of Homer-binding, the Cortactin-binding, and the SAM domains of the protein, which are critical for *SHANK3* interactions with other PSD proteins. Homer-binding domain of *SHANK3* binds to the group 1 metabotropic glutamate receptors, such as mGluR1/5 [29]. Functional studies have found impairments in hippocampal synaptic transmission and plasticity in an exon 21 deletion (coding for the Homer binding domain) mouse model [30]. SAM domain of *SHANK3* has been shown be crucial for postsynaptic localization [31]. Two patients (P13 and P15) carry a c.3679dupG which would generate a premature stop codon at position 1227, causing the loss of the Homer-binding, the Cortactin-binding and the SAM domains of the protein. Functional studies on c.3679dupG have been shown to affect neuronal development and decreased growth cone motility [32].

The key symptoms of PMS are speech delay, DD/ID, ASD and hypotonia, which is in accordance with previous studies [32]. We found a high frequency of hyperactivity (83%) and impulsiveness (76%), which have been previously reported in the literature. 100% of children were able to walk but at a delayed age, which is compatible with a different PMS cohort [32]. Lymphedema, hypothyroid, diabetes, vitiligo and enzyme deficiency absent in individuals in our cases have been reported in patients with PMS [5, 22, 23, 27]. Furthermore, there was a remarkable difference in the frequency of certain PMS features (i.e. overheats or turns red easily, gait abnormalities, gastroesophageal reflux, long eyelashes, epicanthal folds and so on) between our cohort and reported cohorts in literatures. These discrepancies could be related to the relatively young age of the individuals in your cohort (mean age = 4.46 years). Aside from that, the fact that no patients have been evaluated in person which represents a major limitation as well as the fact that only a small percentage of the patients have received the same amount of laboratory and imaging tests contributed to the discrepancies.

Comparisons of phenotypes between patients with loss of *SHANK3* alone and that with deletions encompassing more than *SHANK3* gene showed that there was no significant difference between two groups in the clinical features including DD/ID, absence of speech and ASD in accordance with a previous study [19]. We also demonstrated hypotonia frequency was slightly higher in the loss of *SHANK3* alone group. However, hypotonia frequency showed a correlation with the larger deletions in their study. The smallest deletion in their study encompassed *SHANK3, RABL2B* and ACR genes which cannot implicate the role of *SHANK3* alone in hypotonia. In addition, we observed a high frequency of the increased pain tolerance, gait abnormalities, repetitive behaviors, regression, and minor dysmorphic traits including ear anomalies and descending palpebral fissure in loss of *SHANK3* alone group which are in line with previous estimates in individuals with PMS due to *SHANK3* point mutations [28]. There was no significant difference between two groups in these features. These results indicate that *SHANK3* haploinsufficiency affects these important phenotypes. Although their work compared clinical features in individuals with *SHANK3* mutations to 22q13 deletions including *SHANK3* from the literature, they did not analyze genotype–phenotype correlations between two groups. In the present study, Fisher’s exact two-sided test was used to assess statistical significance in important phenotypes between two groups to analyze genotype–phenotype correlations for the first time. Furthermore, 17 patients with *SHANK3* point mutations and 60 individuals with 22q13 deletions from the literature in their study were not the same cohort. Thus, the methods de Rubeis et al. collected data were different from that in the literature leading to information bias. Finally, cases with 22q13 deletions de Rubeis et al. used to compare phenotypes included two patients (SH29 and SH32) who carried *SHANK3* point mutations (c.2497delG and c.1527G > A respectively), so the prevalence of phenotypes in patients with 22q13 deletions were not correct. Several studies have examined genotype–phenotype associations in *SHANK3* deficiency and results are inconsistent. Phenotypes correlated with deletion size in their studies included renal abnormalities, lymphedema, large or fleshy hands, dolichocephaly, facial asymmetry, feeding problems, genital anomalies, head circumference, recurrent ear infections, pointed chin, and dental anomalies. However, only two reports on PMS have consisted of a few patients carrying *SHANK3* mutations or deletions only disrupt *SHANK3* [5, 22]. In addition, these medical comorbidities frequency were all low or absent in two groups in our study probably leading to the insignificance because of the small sample size. Latha et al. found deletion size was statistical significance associated with ASD. Our evaluation of ASD depended on medical record review while their study included evaluations using standard diagnostic scales with ASD (e.g., ADOS-2 and ADI-R).

There were several limitations to this analysis. We obtained medical history by medical record review or questionnaires completed by parents and the collected data may be subject to recall or information bias. Besides, assessments using standard diagnostic scales with ASD and developmental delay were not available, and thus analyses depend on medical record review. The phenotype comparisons identified in our analysis are heavily influenced by sample size, future studies could be done in larger samples to provide a clear role of *SHANK3* haploinsufficiency in the important phenotypes in PMS.

In summary, this is the first detailed report of the comparisons of phenotypes between PMS patients with deletions encompassing many genes including *SHANK3* and loss of *SHANK3* alone*.* Our findings show loss of *SHANK3* alone is sufficient to produce the characteristic phenotypes of PMS, including developmental/neurological abnormalities, behavioral features, gastrointestinal problems and dysmorphic features. We also observed a high frequency of the speech delay, DD/ID, ASD, hypotonia, increased pain tolerance, impulsiveness, repetitive behaviors, regression, nonstop crying, and minor dysmorphic traits including ear anomalies and descending palpebral fissure in loss of *SHANK3* alone group and there was no significant difference between two groups regarding these important features. These findings extend the role of *SHANK3* haploinsufficiency in PMS beyond its well-known role at the neurological features.

## Methods

### Cohort

Twenty nine previously unreported patients with clinical and genetic diagnosis of PMS in Mainland China from the China League of PMS Rare Disease were recruited in this study from 2018 to 2020. This cohort included 20 males (66%) and 10 females (34%), with age ranging from 1.9 to 9 years old (mean = 4.46, SD = 2.18). Parents or guardians provided genetic exams, medical record review and completed a standardized medical history questionnaire. The questionnaire included queries about the individuals’ stature, head circumference, developmental/neurological features, behavioral abnormalities, and additional clinical features. Level of developmental delay, autism diagnosis, MRI information, perinatal events, growth parameters at birth were abstracted from medical record review. In addition to that, 22 families also provided pictures of frontal, bilateral view of the face, and a view with the eyes closed of their affected children. Two clinicians from Xinhua Hospital, School of Medicine, Shanghai Jiao Tong University assessed the pictures of the patients to analyze their morphological features. All the clinical features refer to it at the age of test, except level of developmental delay, autism diagnosis, MRI information, renal abnormities, perinatal events, growth parameters at birth which were abstracted from medical record review.

The study was approved by Ethics Committee of Xinhua Hospital, School of Medicine, Shanghai Jiao Tong University (XHEC-D-2020-072) and parents provided informed consent forms.

### Genetic testing

Twenty patients have a deletion at 22q13.3 encompassing *SHANK3*. Different exams and platforms were applied: array-CGH (n = 2), SNP array (n = 15) or WES (n = 3) were performed in 20 individuals. Three deletions only disrupt *SHANK3* were validated by MLPA or QPCR (see details in Table 1). *SHANK3* mutations were identified by whole exome sequencing (WES) and then validated by Sanger sequencing in nine individuals. Nine genetic tests were conducted by our research team and the others by outsourced laboratories.

### Statistical analysis

Children were divided into two groups to investigate the role of *SHANK3* haploinsufficiency in the important phenotypes. Group 1 (n = 17) included individuals with deletions encompass more than *SHANK3* gene. Group 2 (n = 12) consists of individuals with *SHANK3* mutations or deletions only encompassing *SHANK3*. Fisher’s exact two-sided test was used to assess statistical significance in important phenotypes between two groups. Results were judged to be statistically significant at *P* < 0.05 and of borderline significance at 0.05 < *P* < 0.10.

## Supplementary information

**Additional file 1**. Main clinical features of individuals with PMS and dysmorphic features in Mainland China PMS patients.

## Data Availability

All data are shown in this article.

## Abbreviations

*SHANK3*: SH3 and multiple ankyrin repeat domains 3
PMS: Phelan–McDermid syndrome
OMIM: Online Mendelian inheritance in man
DD: Developmental disability
ID: Intellectual disability
ASD: Autism spectrum disorder
SD: Standard deviation
Array-CGH: Microarray-based comparative genome hybridization
SNP-array: Single nucleotide polymorphism array
WES: Whole exome sequencing
MLPA: Multiplex ligation probe amplification
QPCR: Real-time quantitative PCR detecting system
Kb: Kilobase
Mb: Megabase
EVS: Exome variant server
gnomAD: Genome aggregation database
ACMG: American College of Medical Genetics and Genomics
EEG: Electroencephalography
MRI: Magnetic resonance imaging
mGluRs: Metabotropic glutamate receptors
ADOS-2: Autism diagnostic observation schedule, second edition
ADI-R: Autism diagnostic interview-revised
