# Supplementary material for: A 29 Mainland Chinese cohort of patients with Phelan–McDermid syndrome: genotype–phenotype correlations and the role of SHANK3 haploinsufficiency in the important phenotypes
Source: Orphanet J Rare Dis. 2020 Nov 30;15:335. doi: 10.1186/s13023-020-01592-5 (PMC7708101; doi:10.1186/s13023-020-01592-5)
Supplement: Supplementary file 1 — Additional file 1. Main clinical features of individuals with PMS and dysmorphic features in Mainland China PMS patients. [file 13023_2020_1592_MOESM1_ESM.docx]

## Additional file

**Table S1 Main clinical features of individuals with PMS.** + present, - absent, *NA* not applicable, *NK* not known, *F* female, *M* male, *ID* intellectual disability, *DD* developmental disability, *MRI* magnetic resonance imaging, *EEG* electroencephalography.

| **Patient** | **Sex** | **Birth related features** | | | **Age**  **at test**  **(year)** |  | **Growth** |  |  | **Development/Neurological features** | | | | | | | |
| --- | --- | --- | --- | --- | --- | --- | --- | --- | --- | --- | --- | --- | --- | --- | --- | --- | --- |
|  |  | **Gestational age (weeks)** | **Birth weight**  **(kg)** | **Birth length**  **(cm)** |  | **Weight**  **(kg, percentile)** | **OFC**  **(cm, percentile)** | **Height**  **(cm, percentile)** |  | **Sat independently**  **(mo)** | **Walked independently**  **(mo)** | **Raising head (mo)** | **Standing independently (mo)** | **First words and current language ability** | **DD/ID (age)** | **Gross**  **motor delay (age)** | **Fine motor delay (age)** |
| P1 | M | 30.3 | 1.4 | 40 | 2.3 | 12(20) | NK | 90(30) |  | 11 | 13 | 15 | 14 | No words | Normal ID (2.3y) | Mild (2.3y) | Mild (2.3) |
| P2 | M | 37.7 | 3.4 | 50 | 1.9 | 16(>97) | 49(73) | 94(97) |  | 7 | NA | 2 | No standing | Single words or phases | Mild ID (1.2y) | Mild (1.2y) | Mild (1.2) |
| P3 | M | 37.7 | 3 | 48 | 5.5 | 21(60) | 52(75) | 115(50) |  | 19 | 33 | 3 | 16 | No words | Mild ID (2.3y) | Mild (2.3y) | Mild (2.3) |
| P4 | F | 35 | 2.5 | 49 | 6.5 | 20(25) | 48(1) | 115(17) |  | 7 | 23 | 3 | 12 | Single words or phases | + (6.5y) | + (6.5y) | + (6.5) |
| P5 | M | 41 | 4 | 52 | 5.8 | 24(90) | 58(90) | 120(90) |  | 11 | 34 | 8 | 30 | No words | + (5.8y) | + (5.8y) | + (5.8) |
| P6 | F | 39.4 | 2.7 | 48 | 1.8 | 12.6(75) | 46(60) | 90(95) |  | 8 | NK | 4 | NK | No words | + (1.8y) | Extremely severe (1.8y) | Extremely Severe (1.8) |
| P7 | M | 39 | 3.7 | 51 | 8.6 | 26(25) | 53(90) | 130(50) |  | NK | 16 | 3 | NK | No words | Extremely severe ID (6.9y) | Severe (6.9y) | Extremely severe (6.9) |
| P8 | M | 37 | 3 | 52 | 3.7 | 15(17) | 52(96) | 100(25) |  | 11 | 27 | 8 | 14 | No words | NK | NK | NK |
| P9 | M | 38.3 | 3.4 | 50 | 7.3 | 28(75) | 54(95) | 124(37) |  | 6 | 14 | 2 | 10 | Single words or phases | Normal ID (3.8y) | Normal (3.8y) | Mild (3.8) |
| P10 | M | 40 | 3.8 | 50 | 6.0 | 20(10) | NK | 113(17) |  | 19 | 15 | 4 | 13 | Single words or phases | + (6.0y) | Severe (6.0y) | Yes (6.0) |
| P11 | M | 38.4 | 4 | 55 | 5.5 | 23(82) | NK | 116(60) |  | 6 | 14 | 3 | 13 | Single words or phases | + (5.5y) | + (5.5y) | + (5.5) |
| P12 | M | 41.3 | 3.3 | 33 | 1.5 | 11.5(54) | NK | 80(19) |  | NK | 17.5 | 3 | 16 | No words | + (5.1y) | Mild (5.1y) | Extremely severe (5.1) |
| P13 | M | 39.3 | 6.2 | NK | 7.1 | 23(37) | NK | 125(53) |  | 7 | 14 | 4 | 11 | Sentences | Mild ID (4.6y) | Normal (4.6y) | NK |
| P14 | M | 40 | 3.2 | 51 | 3.1 | 15(60) | NK | 98(55) |  | 8 | 16 | 2 | 14 | No words | + (3.2y) | + (3.2y) | + (3.2) |
| P15 | M | 29 | 2.95 | 52 | 5.7 | 22.2(75) | NK | 118(32) |  | 8 | 13 | 3 | 12 | No words | Mild ID(2.8y) | Normal (2.8y) | NK (2.8y) |
| P16 | F | 27 | 2.98 | 50 | 4.8 | 20(97) | 53(97) | 110(97) |  | 12 | 33 | 5 | 18 | Single words or phases | + (1.8y) | + (1.8y) | + (1.8y) |
| P17 | M | 40.3 | 3.35 | 50 | 6.4 | 23(25) | 49.5(1) | 117(50) |  | 10 | 22 | 3 | 20 | Single words or phases | Severe ID (6.5y) | Severe (6.5y) | NK (6.5y) |
| P18 | M | 37.3 | 2.9 | 50 | 2.2 | 14(37) | NK | 94(25) |  | 8 | 18 | 3 | 14 | Single words or phases | + (2.1y) | + (2.1y) | + (2.1y) |
| P19 | F | 37.3 | 2.5 | 49 | 1.7 | 9.8(10) | 44(<1) | 83(32) |  | 14 | NA | 7 | NK | No words | Moderate ID (0.5y) | Severe (0.5y) | Moderate (0.5y) |
| P20 | M | 37.3 | 3.2 | 50 | 4.7 | 15.2(3) | 48(12) | 107.5(26) |  | NK | 14 | 5 | NK | No words | NK (4.6y) | NK (4.6y) | NK (4.6y) |
| P21 | F | 37.3 | 2.585 | 50 | 4.3 | 33(62) | 48(10) | 108(90) |  | 7 | 16 | 3 | 12 | No words | Mild ID (1.7y) | Normal (1.7y) | Mild (1.7y) |
| P22 | M | 38.3 | 2.9 | 50 | 5.0 | 20.3(73) | 52(90) | 116(81) |  | 8 | 14 | 5 | 14 | No words | Severe ID (4y) | Moderate (4y) | Severe (4y) |
| P23 | F | 29 | 3.11 | 55 | 3.3 | 17.2(90) | 50(50) | 104.5(95) |  | 5 | 18 | 5 | 16 | Single words or phases | Moderate ID (3.3y) | Mild (3.3y) | Moderate (3.3y) |
| P24 | F | 37 | 3.95 | 53 | 9.0 | 25(25) | NK | 120(3) |  | 12 | 16 | 3 | 12 | Single words or phases | + (9y) | + (9y) | + (9y) |
| P25 | F | 36.7 | 2.73 | 40 | 1.8 | 10.3(12) | 48(79) | 88(80) |  | 21 | NA | 8 | No standing | No worlds | Extremely severe ID (1y) | Extremely severe (1y) | Severe (1y) |
| P26 | M | 41 | 3.2 | 50 | 6.5 | 21.2(25) | 52(53) | 117(25) |  | 8 | 12 | 1 | 12 | No words | NK (6.5y) | NK (6.5y) | NK (6.5y) |
| P27 | F | 39.1 | 3.45 | 50 | 1.4 | 10.3(37) | 51.5(100) | 87(98) |  | 6 | NA | 2 | 16 | No words | Mild ID (1.3y) | Mild (1.3y) | Mild (1.3y) |
| P28 | F | 41 | 3.72 | 54 | 2.7 | 14(62) | 50(91) | 91(25) |  | 10 | 14 | 2 | 22 | No words | + (2.6y) | + (2.6y) | + (2.6y) |
| P29 | M | 38 | 3.35 | 50 | 3.1 | 16(75) | 50(60) | 103(90) |  | 8 | 18 | 3 | 16 | No words | Moderate ID (2.3y) | + (2.3y) | Mild (2.3y) |

| **Patient** | **Development/Neurological features** | | | |  | **Behavioral abnormalities** | | | | | | | | | | |
| --- | --- | --- | --- | --- | --- | --- | --- | --- | --- | --- | --- | --- | --- | --- | --- | --- |
|  | **Language**  **delay (age)** | **Brain MRI (age)** | **Abnormal EEG** | **Seizures** |  | **Decreased perspiration/**  **Heat intolerance** | **Overly sensitive to touch** | **Biting**  **(self or others)** | **Hair pulling** | **Excessive screaming** | **Nonstop crying** **(causes)** | **Self-injury** | **Pica** | **Repetitive behaviors** | **Regression** **(age, details and a trigger)** | **Impulsiveness** |
| P1 | + (2.3) | Generous extracerebral spaces (0.5y) | NK | - |  | - | - | - | + | - | - | - | - | - | - | - |
| P2 | Mild (1.2) | Delayed myelination (0.5y) | + | - |  | - | - | + | + | - | + (no causes) | - | - | + | + (1y; loss of cognitive; no trigger) | + |
| P3 | Mild (2.3) | Thinning of corpus callosum (2y) | + | + |  | - | - | + | - | + | + (no causes) | + | - | + | - | + |
| P4 | NK (6.5) | NK | - | - |  | - | - | - | - | - | - | - | - | - | - | + |
| P5 | + (5.8) | Agenesis of corpus callosum (2.3y) | + | + |  | - | - | - | - | - | - | - | - | - | - | - |
| P6 | NK | Normal | - | - |  | - | + | - | - | - | + (no causes) | - | - | + | - | + |
| P7 | Extremely severe (6.9) | Delayed myelination (2y) | - | + |  | - | + | + | + | + | +(seizure) | - | + | + | + (3y; loss of language; seizure) | + |
| P8 | + (3.7) | Thinning of corpus callosum (0.5y) | NK | + |  | - | - | + | - | + | - | - | - | + | + (2y; loss of language and cognitive; pneumonia) | + |
| P9 | Mild (3.8) | Leukodystrophy (1.5y) | - | - |  | - | - | + | - | - | - | - | - | + | + (1.4; social; fever) | + |
| P10 | + (6.0) | NK | NK | - |  | - | - | - | - | - | - | - | - | + | + (3y; loss of language; no trigger) | + |
| P11 | + (5.5) | Large cisterna magna (4y) | + | - |  | - | + | - | - | - | - | - | - | + | + (4y; loss of language; no trigger) | + |
| P12 | Extremely severe (5.1) | Normal | - | - |  | - | - | - | - | - | - | - | - | - | NK | - |
| P13 | Mild (4.6) | Normal | - | - |  | - | - | + | - | - | - | - | - | + | - | + |
| P14 | + (3.2) | NK | NK | - |  | - | - | + | - | + | + (ear infection) | - | - | + | + (2y; loss of language and cognitive; no trigger) | + |
| P15 | Moderate (2.8y) | NK | - | - |  | - | - | - | - | - | - | - | - | - | - | - |
| P16 | Mild (1.8y) | Enlargement of ventricles (0.5y) | - | - |  | + | - | - | - | - | - | - | - | - | - | - |
| P17 | Extremely severe (6.5y) | Normal | - | - |  | - | - | - | - | - | - | - | + | + | - | + |
| P18 | Moderate (2.1y) | NK | NK | - |  | - | - | + | - | - | - | - | - | - | - | + |
| P19 | Moderate (0.5y) | Enlargement of ventricles (0.5y) | - | - |  | - | - | - | + | - | + (gastroesophageal reflux) | - | + | + | - | + |
| P20 | NK (4.6y) | Normal | NK | - |  | - | - | - | - | + | + (no causes) | + | - | + | - | + |
| P21 | Severe (1.7y) | NK | - | - |  | - | - |  | + | - | + (no causes) | - | - | + | + (3y; loss of language and cognitive; no trigger) | + |
| P22 | Extremely severe (4y) | Normal | + | - |  | - | - | - | + | - | + (no causes) | - | - | - | + (1y; loss of language; fever) | + |
| P23 | Severe (3.3y) | Normal | NK | - |  | - | - | - | - | - | - | - | - | - | + (3y; loss of language and cognitive; fever) | - |
| P24 | + (9y) | Normal | - | - |  | - | - | - | - | - | + (no causes) | - | - | + | + (4y; loss of language and cognitive; change of a caregiver) | + |
| P25 | Severe (1y) | Thinning of corpus callosum (1.6y) | - | - |  | - | + | - | - | - | - | - | - | - | - | - |
| P26 | NK (6.5y) | Normal | + | + |  | - | - | - | + | - | + (no causes) | - | - | - | + (2y; loss of language and cognitive; no trigger) | + |
| P27 | Moderate (1.3y) | Generous extracerebral spaces (1y) | - | - |  | - | - | + | + | - | + (no causes) | - | - | + | - | + |
| P28 | + (2.6y) | NK | NK | - |  | - | - | - | - | - | - | - | - | + | - | + |
| P29 | Severe (2.3y) | Large cisterna magna (2.3y) | NK | - |  | + | + | + | + | - | + (no causes) | - | - | + | - | + |

**Table S1 Main clinical features of individuals with PMS*(Continued).*** + present, - absent, *NA* not applicable, *NK* not known, *F* female, *M* male, *ID* intellectual disability, *DD* developmental disability, *MRI* magnetic resonance imaging, *EEG* electroencephalography

| **Patient** | **Behavioral abnormalities** | | | | | | | |  | **Gastrointestinal problems** | | |  | **Additional features** | | | | | | | | |
| --- | --- | --- | --- | --- | --- | --- | --- | --- | --- | --- | --- | --- | --- | --- | --- | --- | --- | --- | --- | --- | --- | --- |
|  | **Hypotonia** | **Aggression** | **Gait abnormalities** | **Increased pain tolerance** | **Overheats or turns red easily** | **Arachnoid cyst** | **Chewing difficulties** | **Sleep disturbance** |  | **Gastroesophageal reflux** | **Constipation** | **Diarrhea** |  | **Genital anomalies** | **Immune deficiency** | **Recurring upper respiratory tract infections** | **Renal abnormalities** | **Congenital heart defect** | **Allergies** | **Asthma** | **Eczema** | **Hearing loss** |
| P1 | + | - | + | - | - | - | - | - |  | - | - | - |  | + | - | - | - | - | - | - | - | - |
| P2 | + | + | - | - | + | - | - | - |  | - | - | - |  | - | - | + | - | - | - | - | - | - |
| P3 | + | + | + | + | - | - | - | - |  | + | - | - |  | - | - | - | + | - | - | - | - | - |
| P4 | - | - | + | + | - | - | + | + |  | - | - | - |  | - | - | - | - | - | - | - | - | - |
| P5 | + | - | + | + | - | + | - | - |  | - | + | - |  | - | - | + | - | + | - | - | - | + |
| P6 | + | + | - | - | - | - | + | - |  | - | + | - |  | - | - | - | - | - | - | - | + | - |
| P7 | + | + | + | + | + | - | + | + |  | - | + | - |  | - | - | - | - | - | - | - | - | - |
| P8 | + | - | + | + | - | + | - | + |  | - | - | - |  | NK | + | + | NK | - | + | + | + | - |
| P9 | - | - | - | + | - | - | - | - |  | + | - | - |  | - | - | - | - | - | - | - | - | - |
| P10 | + | - | + | + | - | - | - | - |  | - | - | - |  | - | - | - | - | - | - | - | - | - |
| P11 | + | - | - | - | - | - | - | - |  | - | - | - |  | - | - | - | - | - | - | - | + | - |
| P12 | + | - | + | + | - | - | + | - |  | - | - | - |  | NK | - | - | NK | - | - | - | + | - |
| P13 | + | + | - | + | - | - | - | - |  | - | - | - |  | - | - | - | - | - | - | - | - | - |
| P14 | + | + | - | + | - | - | - | - |  | - | - | - |  | - | - | - | - | - | + | - | - | - |
| P15 | - | - | - | - | - | - | - | - |  | - | - | - |  | - | - | - | - | - | - | - | - | - |
| P16 | + | - | + | + | - | - | - | - |  | - | - | - |  | - | - | + | + | + | - | - | + | - |
| P17 | + | + | + | - | + | - | + | - |  | - | - | - |  | NK | - | - | NK | - | + | - | - | - |
| P18 | + | - | - | - | - | + | - | - |  | - | - | - |  | - | - | - | - | - | - | - | - | - |
| P19 | + | - | - | - | - | + | + | - |  | - | - | + |  | NK | - | + | NK | + | - | - | + | - |
| P20 | - | + | - | - | - | - | - | - |  | - | - | - |  | - | - | - | - | - | - | - | - | - |
| P21 | + | + | + | + | - | - | - | + |  | - | - | - |  | - | - | - | - | - | - | - | - | - |
| P22 | + | - | + | + | - | - | + | - |  | NK | NK | NK |  | NK | - | - | NK | NK | + | - | - | - |
| P23 | + | - | + | + | - | - | + | + |  | - | - | - |  | NK | - | - | + | - | - | - | + | - |
| P24 | + | + | - | - | - | - | - | - |  | - | + | - |  | - | - | - | - | - | - | - | - | - |
| P25 | + | - | - | + | - | + | + | - |  | - | + | - |  | - | - | - | - | + | + | - | - | - |
| P26 | + | - | + | + | - | - | + | + |  | - | - | - |  | NK | - | - | NK | - | - | - | - | - |
| P27 | + | - | + | - | + | - | - | + |  | - | - | - |  | NK | - | - | NK | - | - | - | - | - |
| P28 | - | - | - | - | - | - | - | - |  | - | + | - |  | - | - | - | - | - | - | - | + | - |
| P29 | + | - | + | + | + | - | - | - |  | - | - | - |  | - | - | + | - | - | - | - | - | - |

**Table S1 Main clinical features of individuals with PMS*(Continued)*** + present, - absent, *NA* not applicable, *NK* not known, *F* female, *M* male, *ID* intellectual disability, *DD* developmental disability, *MRI* magnetic resonance imaging, *EEG* electroencephalography.

**Table S2. Dysmorphic features in Mainland China PMS patients.** + present, - absent.

| ID | P28 | P7 | P10 | P11 | P12 | P13 | P15 | P20 | P23 | P27 | P16 | P9 | P24 | P30 | P4 | P6 | P3 | P5 | P2 | P1 | P26 |
| --- | --- | --- | --- | --- | --- | --- | --- | --- | --- | --- | --- | --- | --- | --- | --- | --- | --- | --- | --- | --- | --- |
| Microcephaly  (< 3rd percentile) | - | - | - | - | - | - | - | + | - | - | - | - | - | **-** | + | - | - | - | - | - | - |
| Macrocephaly  (>98th percentile) | + | - | - | - | - | - | - | - | - | - | - | - | - | **-** | - | - | - | - | - | - | - |
| Sparse eyebrows | - | - | - | - | - | - | - | + | - | + | - | - | - | **-** | - | + | - | - | - | - | - |
| Long eyelashes | - | - | - | - | - | - | - | - | - | - | - | - | - | **-** | - | - | - | - | - | - | + |
| Periorbital fullness | - | - | - | - | - | - | + | + | - | - | - | - | - | **+** | + | + | - | - | - | - | - |
| Descending palpebral fissure | + | + | + | - | - | - | - | - | - | - | - | + | - | **+** | - | + | - | - | + | - | - |
| Hypertelorism | - | - | - | - | - | - | - | - | - | - | - | - | - | **+** | + |  | - | - | - | + | - |
| Strabismus | - | + | - | - | - | + | - | - | - | - | - | + | - | **+** | - | - | - | - | + | - | + |
| Epicanthal folds | - | - | - | - | - | + | - | - | - | - | - | - | - | **-** | - | - | - | - | - | - | - |
| Wide nasal bridge | - | + | - | - | - | - | - | - | - | - | - | - | - | **-** | + | + | - | - | - | - | - |
| Large/wide nose | - | - | - | - | - | - | - | - | - | - | - | - | - | **-** | + | + | - | - | - | - | - |
| Bulbous nose | - | - | - | + | - | - | + | - | - | - | - | - | - | **-** | - | - | - | - | + | - | - |
| Anteverted nares | - | - | - | - | - | + | - | - | - | - | - | - | - | **-** | - | - | - | - | - | - | - |
| Full cheeks | + | - | - | - | - | - | - | - | - | - | - | - | - | **-** | - | - | - | - | - | - | - |
| Frontal bossing | - | - | - | - | - | - | + | - | + | - | - | - | + | **-** | - | - | - | - | + | - | - |
| Short philtrum | - | - | - | - | - | - | - | - | - | - | - | - | - | **-** | - | - | + | - | - | - | - |
| Ear anomalies | - | - | + overfolded helix | +fleshy ears | + low set ears | - | - | + helix, crus, under-development | - | - | - | - | - | **-** | - | + preauricular fistula | - | - | - | - | - |
| Thick lower lip | - | - | + | - | - | - | + | - | - | - | - | - | - | **-** | + | - | - | - | - | - | - |
| Down-turned mouth | - | - | - | - | - | - | - | - | + | - | - | - | - | **-** | - | - | + | + | - | - | - |
| Short stature/delayed growth  (< 3rd percentile) | - | - | - | - | - | - | - | - | + | - | - | - | - | **-** | - | - | - | - | - | - | - |
| Tall stature/accelerated growth  (>98th percentile) | + | - | - | - | - | - | - | - | - | - | - | - | - | **-** | - | + | - | - | + | - | - |
